# Attitudes and Willingness to Participate in Drug Clinical Trials Among Patients With Cancer: Multistage Qualitative Study

**DOI:** 10.2196/88758

**Published:** 2026-05-05

**Authors:** Sijia Yu, Mengmeng Lyu, Haiping Ma, Keying Xu, Haiyan Li, Lili Ma, Mengting Ji

**Affiliations:** 1 School of Medicine, Tongji University, Shanghai, China; 2 Clinical Research Unit, Renji Hospital, School of Medicine, Shanghai Jiao Tong University, No.160 Pujian Road, Pudong New Area, Shanghai, 200127, China, 86 18817840389; 3 School of Nursing, Shanghai Jiao Tong University, Shanghai, China; 4 Department of Nursing, Shanghai East Hospital, School of Medicine, Tongji University, Shanghai, China

**Keywords:** clinical trials, online health community, participation, attitude, willingness, decision-making, large language models

## Abstract

**Background:**

Cancer clinical trials are essential for advancing therapeutic innovations; however, patient enrollment remains a persistent challenge globally. Understanding the attitudes and willingness of patients with cancer to participate in clinical trials is critical for improving recruitment strategies. While previous studies have explored barriers and facilitators, few have integrated multiple data sources or used emerging analytical approaches, such as large language models (LLMs), to capture the multidimensional nature of patient decision-making. Furthermore, limited research has examined these perspectives within the Chinese health care context, where cultural, economic, and systemic factors may uniquely influence participation decisions.

**Objective:**

This study aimed to examine the attitudes and willingness of patients with cancer to participate in drug clinical trials in China by (1) identifying key themes influencing patients’ decision-making processes, (2) comparing thematic findings derived from investigator-led qualitative analysis with those generated by 2 LLMs (Gemini Pro 2.5 and DeepSeek R1), and (3) evaluating the complementary value of hybrid analytical approaches in qualitative health research.

**Methods:**

A multistage qualitative study was conducted using 2 data sources: semistructured face-to-face interviews with patients with cancer (n=11) from a tertiary hospital in Shanghai and publicly available comments from 2 Chinese online health communities (Zhihu and Yuaigongwu). Of the 3148 initial comments, 219 met the inclusion criteria after systematic screening. Three parallel analytical approaches were used: investigator-led thematic analysis, Gemini Pro 2.5–assisted analysis, and DeepSeek R1–assisted analysis. Both LLMs received identical, structured prompts. Thematic outputs were systematically compared to identify convergent and divergent findings.

**Results:**

The 3 analytical methods jointly identified 7 core themes: treatment selection, financial burden relief, uncertain therapeutic efficacy, uncertainty regarding control groups, lack of cognition, misconceptions, and physician trust. Substantial thematic overlap was observed between investigator-led and DeepSeek R1 analyses (8 shared themes, including family-involved decisions and service-related factors) and between investigator-led and Gemini Pro 2.5 analyses (3 shared themes, including regional disparities and autonomous decision-making). Method-specific themes included recognition of medical value (investigator only), insufficient clinical data (DeepSeek R1 only), and lack of information resource (Gemini Pro 2.5 only). These findings highlight the multidimensional nature of trial participation decisions, encompassing treatment expectations, economic considerations, risk perceptions, cognitive factors, trust relationships, and structural barriers to accessibility.

**Conclusions:**

The willingness of patients with cancer to participate in clinical trials is shaped by a complex interplay of treatment expectations, economic considerations, risk perceptions, cognitive factors, and relational dynamics. The hybrid analytical framework demonstrated complementary strengths: human analysis provided contextual depth and cultural sensitivity, while LLMs offered efficiency and identified additional thematic dimensions. These findings underscore the need for patient-centered communication strategies, transparent trial information, and culturally tailored recruitment approaches. Future research should expand sample diversity and further validate the use of LLMs in qualitative health research.

## Introduction

Cancer is a major cause of death in the developed world. According to GLOBOCAN 2022 data, China recorded 4.825 million new cancer cases and 2.574 million cancer-related deaths annually, accounting for 24% and 26% of the global totals, respectively [1]. This not only imposes a heavy disease burden but also incurs huge socioeconomic costs. Clinical trials are a key means to advance cancer treatment, improve survival rates, and introduce innovative therapies [2]. However, implementing clinical trials faces systemic barriers. More than 80% of trials fail to complete the required sample enrollment within the target timeframe [3,4]. As many as 19% of clinical trials are terminated due to insufficient enrollment [5]. The participation rate among adult patients is extremely low, ranging from 2% to 8% over the long term [6-8]. These deficiencies often require adjustments, such as extending the time frame and increasing the number of test sites, to scale-up participant recruitment. However, these measures have significant financial implications [9].

Patients’ decisions regarding trial participation may be influenced by multidimensional factors, including limited knowledge, concerns about the informed consent process, and low motivation [10-12]. Previous research has extensively explored the factors influencing the decisions of patients with cancer to participate in drug clinical trials. A notable systematic review in 2015, which encompassed 36 quantitative and 15 qualitative reports, identified a broad spectrum of personal, structural, and social factors affecting participation in radiotherapy, surgical, and drug trials [13]. More recently, research has focused specifically on the nuanced context of drug trials. For instance, a 2019 qualitative synthesis of 9 studies found that patient decisions are critically shaped by interpersonal factors, such as trust in physicians, relatives’ attitudes, and perceived impact on their family [14]. However, a key limitation of these studies is their reliance on a single methodological approach, either exclusively qualitative or exclusively quantitative. Previous quantitative studies have examined associations among factors but often fail to capture patients’ subjective experiences. In contrast, qualitative research provides valuable insights into emotional and experiential dimensions, yet small and unrepresentative samples constrain its findings. To overcome these limitations, a mixed methods approach that integrates online discourse analysis with in-depth interviews is warranted, thereby enhancing representativeness and depth of understanding of patient perspectives.

A significant challenge in social and health research is sourcing data that is both authentic in depth and representative in scope. Although qualitative research does not aim for statistical representativeness, the sample’s diversity directly influences theoretical saturation and conceptual coverage. Online data collection enables the rapid construction of a diverse sample pool by allowing algorithmic screening of user content across various geographic, age, and professional backgrounds. Prior studies have modeled patients’ social support needs using data from online health communities (OHCs) to better address their needs [15]. A meta-synthesis of 28 qualitative studies further delineated the opportunities and challenges associated with the use of online social platforms for individuals navigating pregnancy loss [16]. Therefore, this study harnesses the broad scope and authentic depth of online data to investigate the attitudes and willingness of patients with cancer to participate in drug clinical trials.

Large language models (LLMs) have emerged as powerful tools for processing and analyzing textual data. These artificial intelligence (AI) systems, such as GPT, demonstrate capabilities in pattern recognition and theme extraction that can complement traditional qualitative methods [17]. However, LLMs are not entirely neutral analytical tools; they carry biases originating from their training data and algorithmic design. Moreover, LLMs struggle to discern emotional nuances and contextual depth [18]. Human researchers possess a more holistic orientation and deeper understanding, as emphasized by humanism, in complex causal reasoning, theoretical construction, and knowledge production. Thus, this study advocates leveraging LLMs’ current qualitative text-analysis capabilities to assist researchers, thereby enhancing the reliability and reproducibility of research.

Two data collection methods were employed in this study: conducting one-on-one, semistructured interviews and collecting online discourse using Python (Python Software Foundation). In this study’s analytical approach, traditional qualitative analysis is integrated with LLM capabilities. By analyzing patient narratives from these 2 methodological perspectives, this study aims to comprehensively evaluate the attitudes and willingness of patients with cancer to participate in trials, ultimately informing evidence-based strategies to optimize recruitment. The goal is to propose targeted intervention strategies to improve the clinical trial experience for patients with cancer and to provide a scientific basis for facilitating the smooth progression of participant recruitment.

## Methods

### Overview

**Figure 1.**
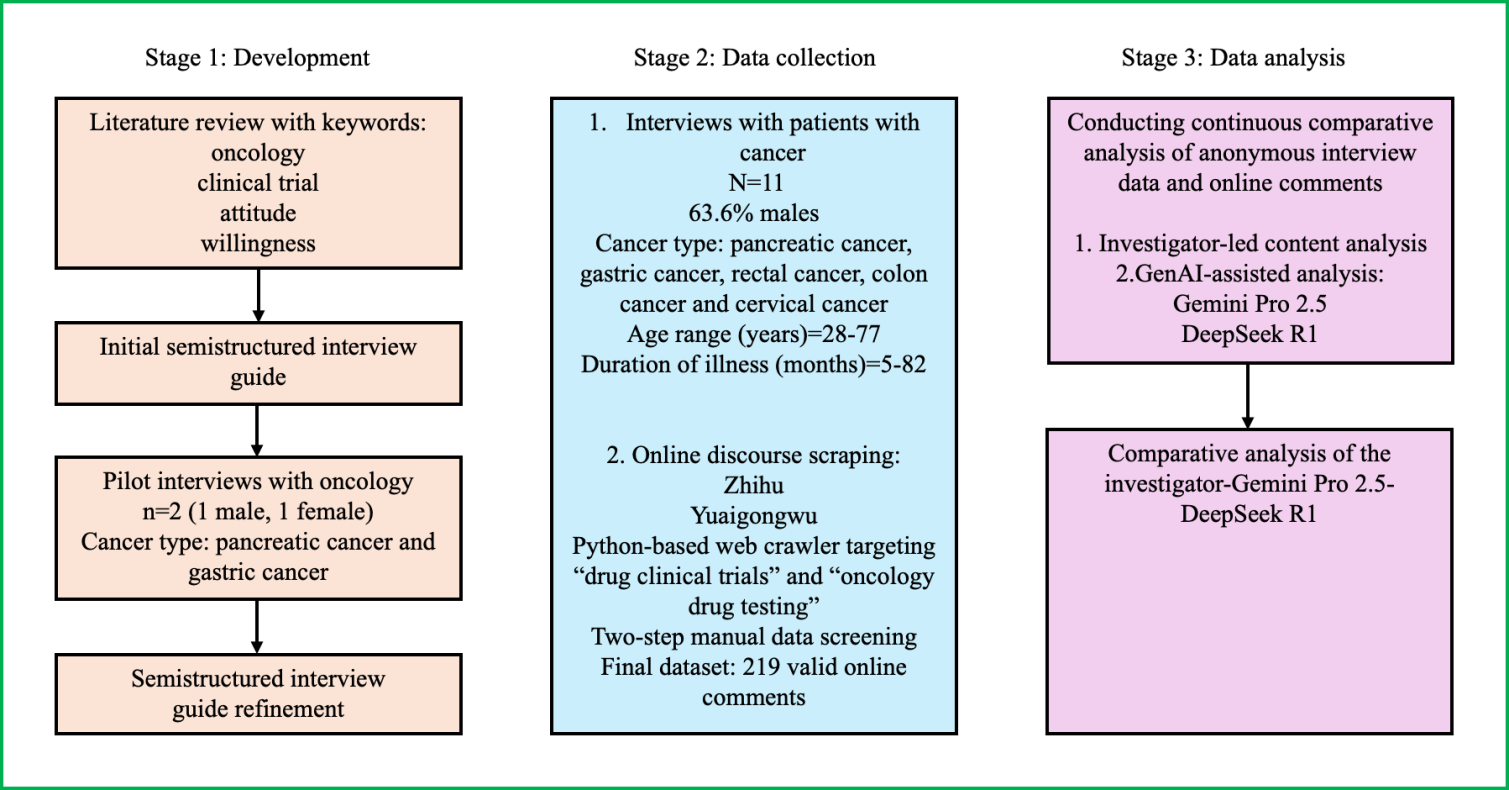
Multistage study design flowchart illustrating the 3 stages of data collection and analysis for exploring the attitudes of patients with cancer toward drug clinical trial participation in China. GenAI: generative artificial intelligence.

This study used a multistage qualitative design that uses 2 distinct data sources examined through 3 analytical lenses. The research process was structured into 3 stages to ensure a comprehensive evaluation. Stage 1 involved preparatory work, specifically the development of the interview guide based on a literature review. Stage 2 comprised dual-channel data collection, obtaining detailed narratives from one-on-one patient interviews and scraping online discourse using Python. Stage 3 focused on analysis and comparative synthesis, where traditional human qualitative coding was triangulated with LLM-driven thematic analysis (Figure 1).

### Interview Guide Development

The initial interview guide was developed from a literature review and refined after pilot interviews, yielding a final version comprising 4 dimensions: personal experiences with clinical trials, decision-making factors, social influences, and willingness to participate in the future (Textbox 1).

Textbox 1. **The final semistructured interview guide.**

Could you please elaborate on your understanding of clinical trials?

- Have you ever participated in a clinical trial?
  - If you have participated in a clinical trial:
    - What type of clinical trial did you participate in?
    - Please describe the process of your participation in the clinical trial and provide an evaluation of it.
    - What were the attitudes of those around you toward your participation?
    - Would you be willing to participate in a new clinical trial? Why or why not?
  - If you have not participated in a clinical trial.
    - What were the reasons for your nonparticipation or refusal to participate in a clinical trial?
    - Would you be willing to participate in a clinical trial? Why or why not?
- Have individuals in your social circle participated in clinical trials? What do you believe were the reasons for their participation or refusal?

### Inclusion and Exclusion Criteria

#### Interview Participants

Purposive sampling was used to recruit patients meeting the inclusion criteria: (1) adults with a histologically or cytologically confirmed malignant solid tumor, (2) an education level of at least junior high school, (3) Eastern Cooperative Oncology Group performance status of 0‐1, and (4) estimated life expectancy of 12 weeks or more. These criteria were established to ensure that participants had sufficient physical stamina to complete 20-40–minute interviews and the cognitive capacity to articulate complex factors in clinical trial decision-making. It is acknowledged that these criteria inherently exclude the most frail patients and those with limited educational backgrounds, and that their perspectives may therefore be underrepresented in this study. All participants were required to participate in the study voluntarily, demonstrate adequate comprehension of the study procedures, and provide written informed consent. Patients were excluded if they had a history of psychiatric disorders, cognitive impairment, communication barriers, or active central nervous system metastases (eg, cerebral edema and progressive lesions). After fully explaining the study’s purpose and procedures to all participants, informed consent was obtained. A total of 11 participants were recruited for the face-to-face interviews.

#### Online Data

Inclusion criteria are (1) comments explicitly discussing cancer drug clinical trials, (2) comments from patients with cancer or their caregivers, and (3) sufficient content length (≥10 Chinese characters) for meaningful analysis.

Exclusion criteria are (1) duplicate comments, (2) incomplete or incomprehensible content, and (3) advertisements or promotional information.

### Data Collection

Data collection integrates individual perspectives and population-level insights. This dual-pronged approach was actualized through in-depth, one-on-one interviews, which provided nuanced personal narratives, and the systematic collection of public comments from online platforms, which offered insights into collective sentiments and spontaneous reactions.

#### Face-to-Face Interviews

Face-to-face interviews were conducted in January 2025. All interviews were conducted by a trained qualitative researcher with experience in clinical trials and oncology care. Each patient was interviewed once; no repeat interviews were conducted. Interviews were scheduled at times convenient for the interviewees and conducted one-on-one sessions in quiet conference rooms, lasting 20 to 40 minutes, to allow participants to independently determine the extent of their disclosures. All interviews were audiorecorded with participants’ informed consent.

#### Online Data Scraping and Screening

For this study, online data were collected via web crawlers, and comments published between September 2010 and May 2025 were retrieved. The textual data originated from 2 distinct Chinese online platforms: Zhihu and Yu-Ai-Gong-Wu (Dancing with Cancer). The selection of these 2 platforms was deliberate, aiming to encompass a wide range of viewpoints by combining extensive public discussions with personal accounts that closely address patients’ needs.

Zhihu is a prominent question-and-answer–based social platform in China, known for its high-quality, in-depth content from a diverse user base of professionals, experts, and engaged laypersons. The total number of views on cancer-related topics on Zhihu has exceeded 3.81 billion. Yuaigongwu, which translates to “Dancing with Cancer,” is a dedicated online support community for patients with cancer and their families, established in 2010. The platform facilitates peer-to-peer support and the sharing of firsthand treatment experiences and emotional journeys. The forum has more than 1 million posts and a membership exceeding 180,000.

Data collection was performed using a custom Python web crawler. The crawler was programmed to scrape publicly accessible text data (posts and comments with timestamps) from the selected platforms by targeting specific keywords, including “drug clinical trials” and “oncology drug testing.” All data were anonymized to protect user privacy, and the collection process complied with relevant laws and the platforms’ terms of service. A rigorous multistage data preprocessing protocol was subsequently implemented to ensure the dataset’s quality and relevance.

A total of 3148 comments related to cancer clinical trials were identified through web crawling. After removing 39 duplicate comments, 3109 unique comments were retained for screening. In the initial screening stage, comments were excluded if they (1) contained advertisements or promotional information (n=75), (2) were unrelated to cancer clinical trials (n=2475), or (3) contained fewer than 10 meaningful characters after removing emojis and hyperlinks (n=208). A total of 2758 comments were excluded, and 351 comments were retained for further eligibility assessment.

In the second-stage eligibility assessment, 2 researchers independently reviewed the remaining 351 comments. Comments were excluded if they were unrelated to the subject of this study (n=118) or were incomplete or unintelligible (n=14). Discrepancies between the 2 reviewers were resolved through discussion and, when necessary, adjudicated by a third researcher. After excluding 132 comments, a final dataset of 219 valid comments was retained for analysis.

Text data were normalized by removing nonessential characters, standardizing punctuation, and tokenizing emoticons that conveyed emotion. All inclusion and exclusion decisions were recorded in a structured dataset that included the post ID, screening results, reasons for exclusion, and reviewer notes. A random 5% of the data was rechecked upon completion to ensure accuracy. This 2-step, transparent screening process ensured the reliability, validity, and reproducibility of the dataset used for qualitative analysis.

### Data Analysis

This study adopts a parallel analytical approach, integrating a researcher-led analysis with an analysis conducted by the LLM. The interview recordings were transcribed using Feishu software (Beijing Feishu Technology Co., Ltd), and 2 researchers independently verified the transcripts for accuracy.

#### Investigator-Led Content Analysis

Content analysis was used to analyze the interview transcripts and online comments separately, thereby distilling key themes. Two researchers independently analyzed the materials by repeatedly reading the textual data, identifying meaningful statements, and assigning codes. Following the summarization, preliminary themes were formulated. In instances of discrepancies during the analysis process, these were resolved through research group discussions until consensus was reached, ultimately determining the final research results.

All interview records were anonymized before analysis to protect confidentiality, especially during the LLM-assisted analysis stage, where special attention was paid to data privacy.

#### Generative AI-Assisted Analysis: Gemini Pro 2.5 and DeepSeek R1

The study required anonymized transcripts to be prepared in plain text format for processing by both Gemini Pro 2.5 (version released by Google AI Studio in March 2025) and DeepSeek R1 (version released in January 2025, accessed via [19]). An initial prompt was developed based on the research objectives and tested on a calibration subset of 20 online comments. The initial prompt used for both LLMs was:

Please analyze the interview records of cancer patients / the online platform comments from cancer patients in the document. Your task is: (1) Identify the main themes and sub-themes related to the patients’ willingness and attitude towards participating in drug clinical trials. (2) For each theme, extract 2 key quotes or phrases that can reflect the theme insights, and explain their significance for understanding the patients’ attitudes.

This calibration phase identified two issues requiring refinement: (1) the initial prompt allowed paraphrased summaries, which hindered quote verification, and (2) output formatting was inconsistent across responses. The prompt was refined to require verbatim quotes and to specify a structured output format. Once finalized, the prompt was fixed and applied consistently to all subsequent analyses without further modification.

Unlike human coders, LLMs do not apply predefined codebooks line by line or engage in iterative coding. Consequently, traditional inter-rater reliability metrics, such as Cohen κ, do not apply to their outputs. Instead, transparency in prompt construction and in human review of AI-generated themes was used to ensure analytic consistency and validity [18].

The initial prompt was refined as follows:

Please analyze the following anonymized [interview transcripts / online comments] from cancer patients regarding their attitudes toward participating in drug clinical trials. Your tasks are as follows: (1) Identify the main themes and subthemes related to patients’ willingness and attitudes toward clinical trial participation. (2) For each theme identified, extract exactly 2 verbatim quotes from the source text that best illustrate the theme, and provide a brief explanation of each quote’s significance. (3) Present your findings in a structured format with theme names, subthemes, supporting quotes, and interpretive explanations. Ensure all quotes are copied exactly as they appear in the source text.

These refinements improved the consistency and verifiability of LLM outputs.

Interview transcripts (N=11) were compiled into a single anonymized document with participant identifiers (P01-P11). Online comments (n=219) were organized into a separate document with sequential identifiers (ZH01-ZH53 and YAGW001-YAGW166), with each comment clearly delimited. Documents were uploaded to each LLM platform separately as contextual input, and the models were prompted with structured instructions to identify overarching themes through in-context learning.

LLM outputs were independently reviewed by two researchers to verify (1) accuracy of quote attribution (ie, that quoted text actually appeared in the source data), (2) coherence and relevance of thematic categorizations, and (3) absence of fabricated or hallucinated content. Discrepancies were resolved through discussion with a third researcher. Quotes that could not be verified in the source data were excluded from the final analysis.

#### Comparative Analysis

**Figure 2.**
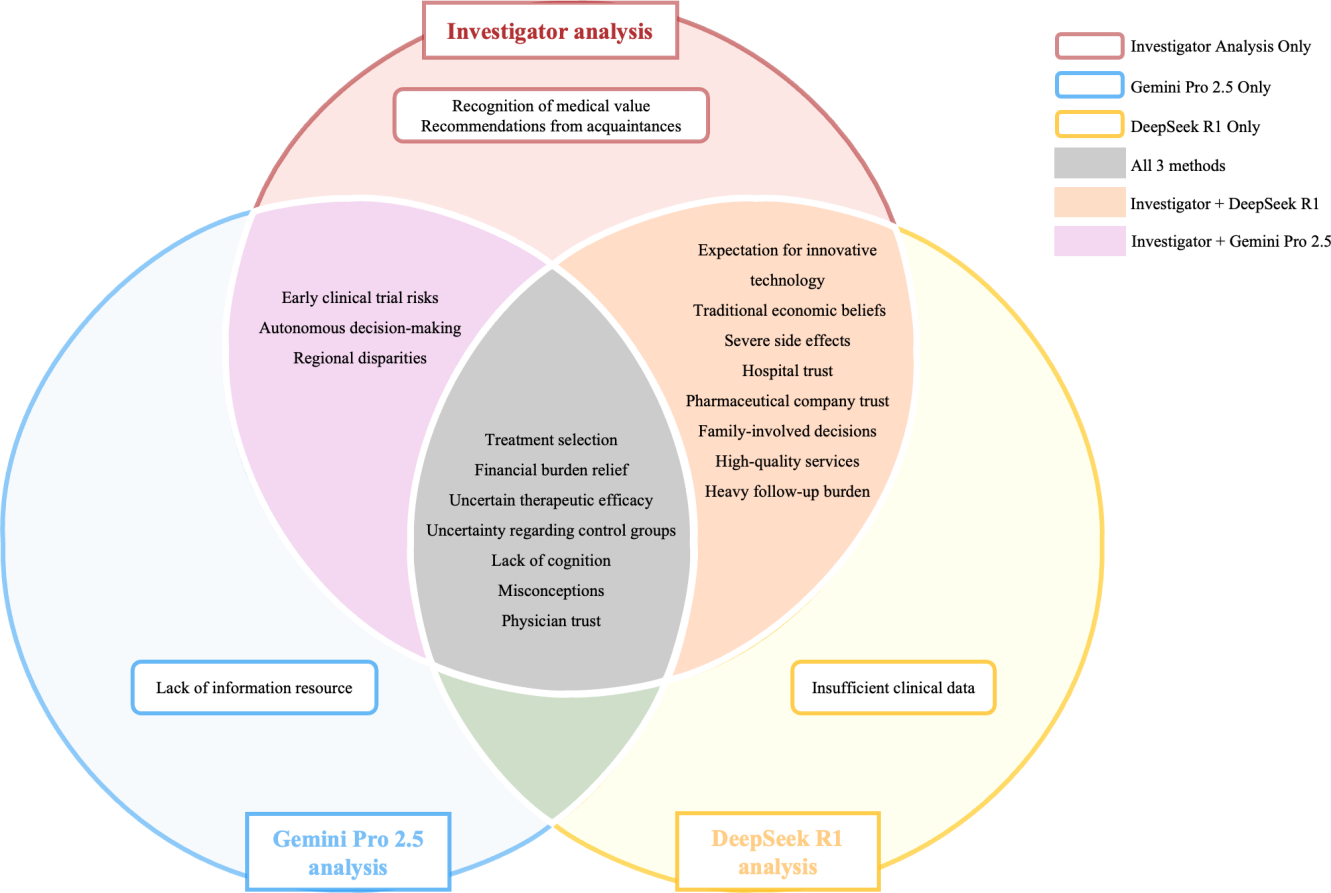
A diagram depicting thematic convergence and divergence across 3 analytical methods (investigator-led, Deepseek R1, and Gemini Pro 2.5) in the analysis of the attitudes of patients with cancer toward clinical trial participation.

To ensure the cross-method reliability of the findings, a systematic comparative analysis was conducted. Themes derived from all analytical methods were evaluated through parallel examination, addressing both terminological variations and interpretive differences. A thematic map was created to link synonymous or related labels, producing a single, integrated thematic framework (Figure 2).

Conceptual alignment was observed when distinct terminologies referenced identical constructs. For instance, the investigator-led subtheme “concerns regarding efficacy,” Gemini Pro 2.5’s “fear stemming from uncertainty,” and DeepSeek R1’s “uncertainty of curative effect” were found to be semantically equivalent and were harmonized into the unified theme of “uncertain therapeutic efficacy.” Conversely, interpretive divergences requiring adjudication occurred when methods captured different intensities or facets of a topic. Rather than treating these as discrepancies, they were leveraged as sources of deeper interpretive insight. A notable discrepancy arose regarding economic motivations: while human analysis identified general “alleviation of economic pressure,” DeepSeek R1 focused on procedural “cost waivers,” and Gemini Pro 2.5 highlighted a more desperate sentiment that “free treatment is the only way out.” The research team re-evaluated the source data and determined that these were distinct but complementary dimensions. Consequently, these were synthesized into the comprehensive theme of “financial burden relief.”

Themes were ultimately classified as convergent if they embodied substantively equivalent ideas across methods. An independent reviewer conducted the classification, a second investigator validated all classifications, and discrepancies were resolved through consensus within the research team.

### Ethical Considerations

This study was approved by the Ethics Committee of Renji Hospital, affiliated with Shanghai Jiao Tong University School of Medicine (LY2025-140-B), and adhered to relevant ethical guidelines, including the Declaration of Helsinki and those of the Association of Internet Researchers [20]. For the face-to-face interviews, written informed consent was obtained from all participants. For the online data component, the ethics committee waived the requirement for individual informed consent, as the research involved a noninterventional analysis of publicly available social media data. We ensured that data collection complied with the platforms’ terms of service regarding publicly accessible content. To strictly protect user privacy, all online data were fully anonymized through a multistep process. First, structured identifiers, such as usernames, were automatically removed during the data scraping phase. Second, 2 researchers manually reviewed the text content to detect and redact any remaining personally identifiable information, such as real names, specific hospital names, and unique location information. This ensured that no quotes presented in this manuscript could be traced back to specific individuals. Each participant received approximately ¥30 RMB (approximately US $4) as transportation reimbursement for their participation in the interviews.

## Results

### Participants of Interviews

**Figure 3.**
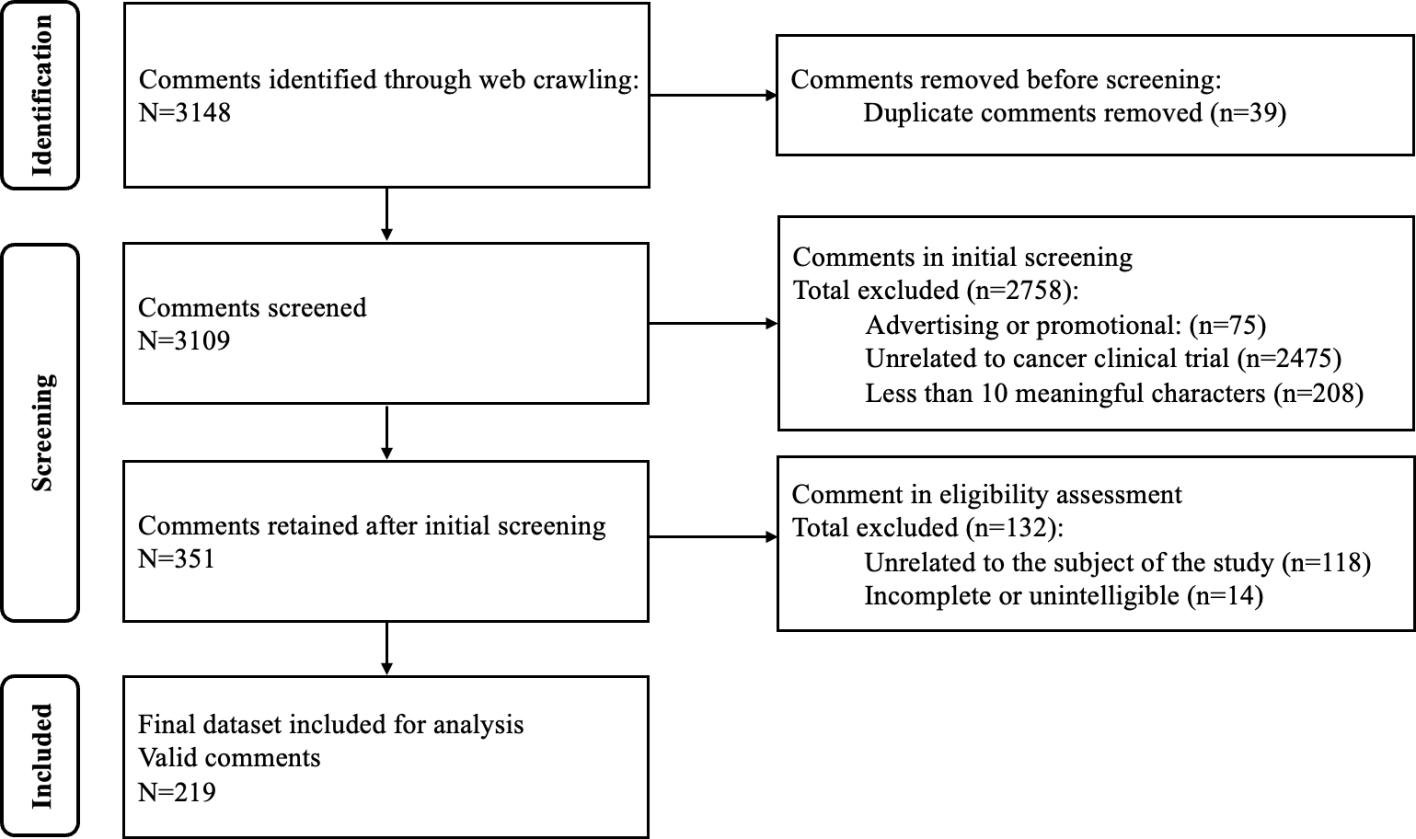
Flowchart of the data collection and screening process related to cancer clinical trials. A total of 3148 comments were retrieved from the online platform and subjected to a 2-stage screening procedure. The final dataset comprised 219 valid comments for analysis.

The interview portion of this study included 11 patients with cancer (n=7 males and n=4 females) with a mean age of 57.73 (SD 13.4; range 28‐77) years. The participants represented a variety of cancer diagnoses, with pancreatic cancer being the most common (n=6, 54.5%), followed by gastric cancer (n=2, 18.2%), and other cancer types (Table 1). The majority of participants were married (n=9, 81.8%), with the remainder reporting other marital statuses. The highest educational attainment among participants was a bachelor’s degree (n=4, 36.4%), while others had completed either high school (n=4, 36.4%) or junior high school education (n=3, 27.3%). All age-related content in this study was entirely derived from the analysis of the interview data (Figure 3).

**Table 1.** Demographic and clinical characteristics of interview participants: patients with cancer recruited from a tertiary hospital in Shanghai, China (N=11). Data collected in January 2025.

Variables	Values
Sex, n (%)	
Male	7 (63.6)
Female	4 (36.4)
Age range (y), mean (SD; range)	57.73 (13.4; 28-77)
Family status, n (%)	
Married	9 (81.8)
Unmarried	1 (9.1)
Widowed	1 (9.1)
Duration of illness (mo), mean (SD; range)	21.82 (19.90; 5-82)
Educational qualifications, n (%)	
Junior high school education	3 (27.3)
High school education	4 (36.4)
Bachelor’s degree	4 (36.4)
Cancer types, n (%)	
Pancreatic cancer	6 (54.5)
Gastric cancer	2 (18.2)
Rectal cancer	1 (9.1)
Colon cancer	1 (9.1)
Cervical cancer	1 (9.1)

### Comments From OHCs

The initial data collection yielded 3148 comments across the 2 platforms. Specifically, 2960 comments were retrieved from Zhihu and 188 from Yuaigongwu. Following the data-cleaning and screening protocol, a final dataset of 219 relevant comments was established, comprising 53 from Zhihu and 166 from Yuaigongwu.

### Themes of Parallel Analysis

The analyses, led by researchers and driven by LLMs, jointly yielded 8 overarching themes (Table 2).

**Table 2.** Thematic coverage across investigator-led and large language model (LLM)-assisted qualitative analysis.

Themes and subthemes	Data source	Analytical method		
		Investigator-led	DeepSeek R1	Gemini Pro 2.5
Treatment motivation				
Treatment selection	Interview and OHC^a^ data	✓	✓	✓
Expectation for innovative technology	Interview and OHC data	✓	✓	
Cost considerations				
Financial burden belief	Interview and OHC data	✓	✓	✓
Traditional economic beliefs	Interview data	✓	✓	
Risk perception				
Severe side effects	Interview and OHC data	✓	✓	
Uncertain therapeutic efficacy	Interview and OHC data	✓	✓	✓
Insufficient clinical data	Interview data		✓	
Early clinical trial risks	OHC data	✓		✓
Uncertainty regarding control groups	Interview and OHC data	✓	✓	✓
Cognitive variation				
Lack of cognition	Interview and OHC data	✓	✓	✓
Misconceptions	OHC data	✓	✓	✓
Recognition of medical value	Interview data	✓		
Brand trust effect				
Physician trust	Interview and OHC data	✓	✓	✓
Hospital trust	Interview data	✓	✓	
Pharmaceutical company trust	OHC data	✓	✓	
Decision maker				
Autonomous decision-making	Interview data	✓		✓
Family-involved decisions	Interview data	✓	✓	
Recommendations from acquaintances	Interview data	✓		
Service processes				
High-quality services	Interview and OHC data	✓	✓	
Heavy follow-up burden	Interview and OHC data	✓	✓	
Resource accessibility				
Lack of information resource	OHC data			✓
Regional disparities	Interview and OHC data	✓		

a OHC: online health community.

#### Theme 1: Treatment Motivation

##### Treatment Selection

For some, participation in clinical trials was driven by treatment failure at later stages, motivated by the pursuit of any remaining survival opportunities:

*The previous trial was stopped because my liver lesions had recurred. The doctor said the drug wasn't working well and asked me to stop and rescreen. If I cannot join this program, I'll still need to switch to another treatment.*

[P10]

*I hope to be assigned to the treatment group, because other drugs are no longer effective—if they still worked, who would want to be a guinea pig?*

[ZH07]

##### Expectation for Innovative Technology

Some participants perceived clinical trials as opportunities to access better treatments and increase their chances of improvement:

*This drug has already gone through an earlier phase of testing, and the feedback was positive. Even without this clinical trial, I would still need regular chemotherapy, but participating might offer me more opportunities.*

[P01]

*For some diseases, participating in new drug trials is the only gamble patients can take.*

[ZH52]

### Theme 2: Cost Considerations

#### Financial Burden Relief

Most participants indicated that their decision to join a clinical trial was influenced by financial considerations, aiming to alleviate the ongoing economic burden of high treatment costs for their families:

*If we had to buy this drug as regular consumers, we couldn't afford it. But by joining the clinical trial, it’s like you only have to pay a one-time fee, and then you receive it for free.*

[P08]

*Without the clinical trial, one course of ADC [Antibody-Drug Conjugate] starts at 250,000. There’s no way I could ever afford that myself.*

[YAGW089]

#### Traditional Economic Beliefs

Some interviewees, despite having sufficient family resources, expressed a desire to preserve them for the next generation, influenced by traditional family ethics emphasizing “intergenerational sacrifice” and “intergenerational transfer of resources.”

*If I don't participate, the medicine I take now costs 200,000 a year. My son would certainly agree to it. But I still want to save a little; I want to leave it for my children.*

[P06]

### Theme 3: Risk Perception

#### Severe Side Effects

Concerns regarding potential adverse drug reactions were noted, with some individuals expressing fears about their ability to tolerate such effects:

*I understand that participating in such trials means the drug will have side effects, and I know I won't be able to endure them. At my age, I simply can't handle it anymore. The medicines used in these clinical trials are domestically developed investigational drugs, and the side effects are extremely severe.*

[P05]

*Some of our fellow patients who participated in the olaparib trial said the side effects were very severe—teeth falling out in chunks, even worse than chemotherapy reactions. Many people withdrew from the clinical trial midway...If it is this painful, is it really necessary to join a clinical trial?*

[YAGW110]

#### Uncertain Therapeutic Efficacy

Findings revealed that doubts about therapeutic efficacy and safety risks contributed to participants’ hesitancy toward clinical trials. Refusal to take unapproved research drugs was often attributed to perceived safety issues and limited technical maturity:

*What worries me most is that the clinical trial may not go well, and I'm not confident about its success. It’s not only about whether the drug works, but whether it will truly benefit my condition.*

[P02]

*The doctor recommended that we participate in the X-396 trial, but we are still hesitant. We really don’t know whether it will be effective or not.*

[YAGW117]

#### Insufficient Clinical Data

Some participants perceived that the clinical data provided to them prior to enrollment were insufficient for informed decision-making, particularly regarding key outcome probabilities such as survival, recovery, and mortality rates:

*Consider the balance of risks and gains: during a clinical trial, what are the chances of survival? The chances of death? The chances of recovery?...such probabilities must be made transparent and thoroughly analyzed.*

[P02]

#### Early Clinical Trial Risks

Early clinical trial risks emerged as a notable concern. Participants expressed greater apprehension toward phase I trials due to unclear side effects, whereas phase III trials with more comprehensive safety and efficacy data were generally viewed as more acceptable:

*In phase I, many side effects remain unclear.*

[YAGW089]

*Usually, I check which phase the trial is in. Phase III is the best.*

[YAGW090]

*Phase III clinical trials are worth considering.*

[YAGW091]

#### Uncertainty Regarding Control Groups

Uncertainty about the randomization process was identified as a concern during the trial:

*I'm afraid of being assigned to the control group, because there is a treatment group and a control group.*

[P09]

*If I join, there’s only a fifty–fifty chance of receiving the actual treatment. The other half would be assigned to the control group.*

[YAGW138]

*In a new drug clinical trial, isn't it divided into treatment and control groups? If I end up in the placebo group, it might actually delay my condition.*

[YAGW160]

### Theme 4: Cognitive Variation

#### Lack of Cognition

Several participants expressed that their limited understanding of clinical trials directly contributed to feelings of uncertainty and apprehension regarding participation:

*I really don't know what the outcome of the trial will be—because, after all, I don't understand these things.*

[P09]

*Because I don’t understand anything right now, there is an instinctive sense of worry about free trials.*

[YAGW025]

#### Misconceptions

Some patients develop misconceptions due to a lack of understanding of clinical trials:

*With investigational drugs, the patient bears the risk. If it succeeds, the credit goes to the inventors, and they get to make big money! Is that fair?*

[ZH48]

*Clinical trials are just using ordinary people’s health for experiments. It’s a way to profit from harming people, and it’s legal, with no accountability.*

[ZH32]

#### Recognition of Medical Value

Several participants recognized that clinical trials provide not only individual treatment opportunities but also hold forward-looking value for the medical field. They emphasized that the effectiveness of real-world data is critical for breakthroughs in disease treatment and for broader progress in health care:

*It gives patients more opportunities, and for the medical field, it represents an exploratory step forward for a particular disease. With the accumulation and application of technology, such exploration might identify better drugs or therapies and eventually upgrade treatment approaches.*

[P01]

*Clinical trials are definitely for patients, to benefit them. They need to be conducted in hospitals or research centers so that the drug can be promoted, allowing patients to access it earlier in the market.*

[P03]

### Theme 5: Brand Trust Effect

For some individuals, the decision to enroll was heavily reliant on trust conveyed through authoritative channels, including institutional credibility and the doctor-patient relationship.

#### Physician Trust

Participants frequently cited trust in their physicians as a key factor in their willingness to participate in clinical trials. The reputation and recommendation of the treating physician provided a sense of reassurance and credibility.

*Just for the name of Director Wu alone—it’s enough to go ahead without hesitation!*

[YAGW014]

*My attending doctor told me about it, and we are willing to listen to the doctor.*

[P08]

### Hospital Trust

Trust in the hospital conducting the trial also played an important role. Participants believed that reputable hospitals would only be involved in rigorous and legitimate research, which increased their confidence in the trial.

*I know that for a drug, especially a cancer drug, to be brought to market and to be selected for clinical research at a hospital like Renji, it must not be just any ordinary drug.*

[P03]

### Pharmaceutical Company Trust

Participants expressed greater willingness to enroll in trials sponsored by well-known pharmaceutical companies. The brand reputation and international recognition of such companies were perceived as indicators of trial legitimacy and safety.

*Merck is a major, internationally renowned company. Its reputation and brand recognition are top-tier; they wouldn't conduct an illegitimate trial.*

[YAGW034]

#### Theme 6: Decision-Maker

##### Autonomous Decision-Making

Certain participants asserted that the decision to participate in a clinical trial was ultimately their own, emphasizing that family members could not make this choice for them:

*Ultimately, the deciding factor in this matter is me.*

[P01]

*Because with something like this, every decision made is one I must take responsibility for myself.*

[P04]

##### Family-Involved Decisions

Some interviewees viewed family consultation as an essential part of the medical decision-making process:

*I would definitely need to discuss it with my wife and child.*

[P02]

*Of course, that was a decision made after discussing it with my family.*

[P08]

##### Recommendations From Acquaintances

Some respondents stated that their participation in the clinical trial was largely motivated by strong encouragement from people they knew personally:

*My cousin is a doctor at the hospital; she was the one who told me there was an available spot.*

[P10]

### Theme 7: Service Processes

#### High-Quality Services

A subset of participants reported positive experiences with the medical team during the clinical trial, noting that the services provided were excellent and that procedures were flexibly adapted to meet their needs:

*The director made arrangements for me, contacting Renji Hospital in Puxi for my examination, and I was admitted directly to the hospital in Pudong the very next day.*

[P06]

*The hospital here sends a text message first, then follows up with a phone call, and also notifies me via WeChat. I don't have to worry about keeping track of it myself; they automatically call me when it’s time.*

[P08]

*Joining this trial is actually quite good, not to mention it saves a considerable amount of money. [I] am basically a VIP in the hospital, with priority access to all kinds of services, and spared from all the cumbersome procedures like queuing, registration, booking appointments, and waiting for beds.*

[YAGW065]

#### Heavy Follow-Up Burden

Participants reported that the frequency of follow-up visits created a significant time burden and physical fatigue, causing practical disruptions to their daily lives:

*It’s just that the blood draws are too frequent. They require me to come back the very next day for several days in a row. It’s quite inconvenient, and I feel rather fatigued during that time.*

[P04]

*The routine monitoring is especially frequent. For the first few months, there were check-ups every single week—all sorts of CT scans. It takes a significant toll.*

[YAGW144]

### Theme 8: Resource Accessibility

#### Lack of Information Resource

Most patients reported no prior exposure to clinical trials, leading to fear rooted in unfamiliarity. Once diagnosed, they faced a lack of formal channels for acquiring reliable information:

*In smaller towns, it’s more a matter of not knowing. The moment they hear it’s an “experiment,” people are scared off immediately.*

[ZH21]

*It’s an information gap. Fear comes from a lack of understanding, and ultimately, it causes you to miss out on many opportunities.*

[ZH27]

#### Regional Disparities

Due to the specialized nature of clinical trials, these medical resources may be inaccessible in some remote regions. As a result, professional knowledge regarding clinical trials is largely concentrated in major cities, leading patients in outlying areas to be wary of and avoid them:

*At that time, in Urumqi, no such program was available.*

[P01]

*In top-tier metropolitan hospitals, like those in Shanghai, patients have greater awareness and a deeper understanding. Once they understand, they can genuinely grasp the potential benefits of a trial, which makes them more willing to participate.*

[ZH21].

### Themes of Comparative Analysis

Figure 2 presents the results of a comparative thematic analysis, illustrating both the overlapping and unique themes identified through 3 distinct analytical approaches: investigator analysis, Gemini Pro 2.5 analysis, and DeepSeek R1 analysis.

A core set of 7 themes emerged as a consensus across all 3 methods. These common themes, shown in the central box, include (1) treatment selection, (2) financial burden relief, (3) uncertain therapeutic efficacy, (4) uncertainty regarding control groups, (5) lack of cognition, (6) misconceptions, and (7) physician trust. The convergence of these themes across human and AI-assisted analyses strengthens confidence in their validity as central concerns for patients.

Beyond this shared thematic foundation, each analytical approach yielded unique insights. The investigator’s analysis was the only method to identify the themes of “recognition of medical value” and “recommendations from acquaintances.” The Gemini Pro 2.5 analysis uniquely identified “lack of information resource.” Similarly, “insufficient clinical data” was a theme exclusively identified by the DeepSeek R1 analysis.

The analysis also revealed partial thematic overlaps between pairs of methods. The themes of “early clinical trial risks,” “autonomous decision-making,” and “regional disparities” were jointly identified by the investigator and Gemini Pro 2.5 analyses.

Eight themes were jointly identified by the investigator-led analysis and DeepSeek R1: “expectation for innovative technology,” “traditional economic beliefs,” “severe side effects,” “hospital trust,” “pharmaceutical company trust,” “family-involved decisions,” “high-quality services,” and “heavy follow-up burden” (Figure 2). This overlap suggests that DeepSeek R1’s analytical patterns aligned closely with human interpretive approaches for these dimensions.

These method-specific findings demonstrate the complementary value of the hybrid analytical approach: human analysis captured nuanced relational and value-based themes, while LLM analyses identified distinct informational and evidentiary concerns.

## Discussion

### Diversity of Perspectives on the Willingness of Patients with Cancer to Participate in Drug Clinical Trials

This study used a multistage qualitative design to provide comprehensive insights into the willingness and attitudes of patients with cancer toward participation in clinical trials. Patients’ decisions are influenced not only by medical factors but also by personal illness trajectories, family expectations, cultural context, and information accessibility [21-23]. The findings of this study underscore the multidimensional nature of patient decision-making.

The identification of unique themes by the investigator, Gemini Pro 2.5, and DeepSeek R1 further demonstrates that no single analytical perspective can fully capture this complexity. Capturing such diversity, therefore, requires methodological pluralism and interpretive integration. While previous studies have explored patients’ motivations and concerns mainly through interviews or surveys [24,25], our findings deepen this understanding by showing how patients continuously negotiate autonomy, trust, and perceived benefits throughout their engagement in clinical research.

In line with emerging evidence from analyses of online health communities [26,27], our results also reveal how informal, peer-based discussions reflect the emotional, social, and informational dimensions that shape decision-making. Together, these insights highlight the need for communication strategies and trial designs that respect relational autonomy, address patients’ specific concerns, and foster more informed and value-aligned participation.

### Enhancing Methodological Rigor Through Hybrid Analytical Approaches

Traditional approaches to multisource textual analysis face challenges related to subjectivity and contextual variability [28-30]. Recent methodological advancements, such as collaborative digital frameworks [31] and the use of LLMs in text interpretation [32-35], have sought to enhance analytic rigor and transparency. Nevertheless, integrating computational and human-led analyses remains essential to balancing efficiency with interpretive depth.

In this study, the combination of investigator-led thematic interpretation with LLM-based analysis facilitated a more comprehensive understanding of patients’ attitudes toward clinical trials. By triangulating findings from semistructured interviews and online health community discourse, and by using investigator insights alongside Gemini Pro 2.5 and DeepSeek R1, the analysis produced credible, contextually nuanced results. These outcomes are consistent with prior evidence that methodological rigor in qualitative research stems not from a single approach but from the integration of complementary strategies that enhance validity and interpretive transparency [31,36,37].

Collectively, these findings highlight that hybrid analytic frameworks can reveal both convergent and divergent dimensions of patient perspectives, enabling a more balanced representation of lived experiences within the broader landscape of clinical trial participation.

### The Key Factors Influencing Patients' Participation in Clinical Trials

#### Therapeutic Necessity

The majority of respondents enrolled in clinical trials only after their existing treatment failed, leaving them with no alternative options. This suggests that patients believe clinical trials are their only hope, consistent with previous research results [38,39]. Therefore, patients’ enrollment decisions are strongly shaped by therapeutic necessity and the perceived opportunity to access otherwise unavailable interventions [40].

For some patients, the hope and persistent belief that they can be cured of their illness help them regain some normalcy in their lives and enable them to cope with the severe prognosis of their condition [41]. Participants frequently described trial enrollment as a last resort, underscoring the critical role of perceived therapeutic benefit in decision-making. The prevalence of therapeutic necessity necessitates ethically nuanced communication. Recruitment materials should clearly delineate research objectives from therapeutic possibilities, acknowledge the experimental nature of interventions, and avoid implicit guarantees of clinical benefits. Risk-benefit discussions should balance empathy with transparency, clearly distinguishing between research aims and potential clinical benefits. To retain these motivated yet vulnerable participants, proactive emotional support, dynamic consent processes, and ongoing individual education are recommended.

#### Trustworthy Physician-Patient Relationships

The physician-patient relationship is a critical determinant of patients’ willingness to participate in clinical trials. Consistent with prior research, strong trust in a physician correlates with greater willingness to participate [38], whereas institutional skepticism tends to heighten patient concerns [42]. However, recent qualitative findings by Murphy et al [43] reveal that such trust can be ethically ambivalent. While it facilitates engagement, it may also compromise informed consent by leading patients to agree to participate out of a desire to please their physicians or to maintain a valued relationship. This underscores the need for a more balanced communication framework that respects emotional dynamics while maintaining ethical integrity. From the physician’s standpoint, this entails moving from the role of educator to that of collaborator, who acknowledges patients’ emotions, encourages open dialogue, and ensures patients feel heard and understood [44]. Such an approach not only fosters authentic trust but also strengthens shared decision-making and mitigates the risk of undue influence. At the same time, it is emphasized that communication strategies must prioritize verifying patient autonomy over merely relying on trust to increase enrollment.

#### The Economic Calculus of Participation

The findings affirm that economic considerations are a powerful component of the decision-making calculus for many patients. This is consistent with studies such as Sun et al [45], which rank free treatment as a significant motivator. However, this economic driver creates a critical paradox when contrasted with research by Kim et al [46], which documents that socioeconomically disadvantaged populations—the very groups expected to be most responsive to financial incentives—are under-represented in US trials. The simplistic explanation of “cultural differences” is insufficient. Instead, this divergence powerfully illustrates that economic incentives are a necessary but not sufficient condition for participation. Our findings, situated in the Chinese context, highlight the potent “pull” of financial relief. Kim et al [46] work, in turn, reveals the formidable structural barriers, such as low health literacy, logistical hurdles, and historical mistrust, that can completely neutralize this pull for vulnerable populations. This synthesis suggests that the efficacy of an economic incentive is profoundly mediated by a patient’s social and structural context. Therefore, for recruitment strategies to be truly equitable, they must move beyond merely offering financial relief and actively dismantle the systemic barriers that prevent vulnerable patients from even considering the opportunity.

#### Procedural Clarity and Logistical Demands

From the perspective of the service process, participants’ willingness to engage in clinical research is often undermined by procedural burdens such as repeated examinations, long waiting times, and frequent sampling. These requirements cause physical fatigue and psychological disengagement, ultimately reducing retention. Prior research has sought to mitigate this issue through logistical optimization, such as streamlining online follow-ups, improving communication interfaces, and implementing virtual trial workflows, digital tools, and wearables [47,48]. These measures often represent incremental refinements to existing site-centered models rather than structural solutions to patient burden. In contrast, emerging approaches such as patient-centric sampling and decentralized clinical trials aim to address the root causes of these burdens by integrating research into daily life [49,50]. However, the key insight from the data is not simply that technology is superior, but that minimizing physical disruption to the patient’s routine is essential for retention. Therefore, efforts to improve participation should prioritize reducing the protocol’s physical and logistical burden, rather than merely digitizing communication within a burdensome framework.

#### The Emotional Calculus of Risk

Consistent with previous studies, participants’ willingness to participate was influenced by perceived trial risks [23]. The concerns expressed centered on potential adverse reactions, arrangements for posttrial care, and, most critically, the trial’s efficacy. This highlights the urgent need for effective communication to explain potential benefits and risks and to address patient concerns [40]. However, this assessment of risk is not a fixed calculation. Emerging evidence indicates that it is a dynamic process, powerfully shaped by the patient’s evolving clinical reality rather than by the mere duration of their illness. Risk tolerance, for example, often increases with disease severity or after other treatments have failed, as the focus shifts toward potential efficacy. Conversely, a personal history of adverse effects can lead to significant risk aversion [51]. This implies that a one-time, upfront disclosure of risks is insufficient. Instead, risk communication should be regarded as a continuous, adaptive process that evolves alongside the patient’s clinical condition and emotional state. Proactive monitoring of patients’ perceptions and regular follow-up discussions can help identify shifting concerns, clarify misunderstandings, and reinforce trust in the research team. Integrating this dynamic communication model into participant management and recruitment frameworks not only enhances ethical compliance but also strengthens patient retention and data reliability, thereby contributing to the overall integrity and success of clinical trials.

#### Patient Perspectives on Protocol Design

Consistent with previous research, the randomized design of clinical trials remains a major barrier to patient participation [52]. Patients prefer that treatment plans be determined by their physicians rather than randomly assigned [24]. Murphy et al [43] noted that this resistance is often compounded by misunderstandings in which patients erroneously believe that physicians control the allocation. However, Wegge-Larsen et al [5] found that, even when patients fully understood randomization, they remained willing to participate to access novel interventions. This contrast suggests that the barrier of randomization is not absolute but context-dependent. For patients with limited therapeutic options, the opportunity to access a new treatment outweighs the uncertainty of randomization. Conversely, when established treatments are available, randomization is more likely to be perceived as a loss of care. Thus, the effective communication of randomization requires addressing valid patient concerns about care equity, rather than treating resistance solely as a lack of comprehension.

#### The Locus of Agency in Decision-Making

This study’s identification of autonomous, family-delegated, and joint decision-making patterns reveals the complex locus of agency in clinical trial participation. These findings reflect not only individual preferences but also the deep-seated cultural and social contexts within which medical decisions are made. While autonomous decision-making reflects a growing awareness of self-determination, the prevalence of family involvement underscores the influence of values that prioritize collective judgment. This observation aligns with broader findings in oncology care. Systematic reviews by Dijkman et al [21] and Tielemans et al [53] confirm that family involvement in treatment decision-making is a widespread phenomenon, observed across North American, European, and Asian contexts, challenging the notion that it is an exception to the rule of individual autonomy. The regulatory mandate for purely independent decision-making [54], while intended to protect the patient, creates significant tension with this reality. Our findings align with prior research showing that for vulnerable patients, including older adults and those from disadvantaged backgrounds, the ideal of pure autonomy is often practically unattainable, with many ceding decision-making to health care professionals [40]. The role of the family further complicates this picture. Research by Kogan et al [22] powerfully illustrates this dynamic, revealing that caregivers in phase 1 trials often feel compelled to silently support the patient’s choice, withholding their own opinions to protect the patient’s sense of agency. This act of “silent support” reveals a critical nuance: family involvement is not always about co-opting the decision, but is often a mechanism for endorsing and enabling patient autonomy within a supportive relational context. Therefore, the direct transplantation of a universal, Western-centric informed consent model is not merely inappropriate; it is misaligned with patients’ and their families’ lived experiences. It fails to recognize what the evidence consistently shows: the decision-making unit is often the patient-family dyad, not the patient in isolation. A more refined understanding of autonomy is needed, one that transcends rigid individualism and embraces a model of relational autonomy. This perspective preserves the ethical foundation of informed consent while acknowledging the meaningful role of family in decision-making. It recognizes autonomy as contextually shaped, validating both independent and family-informed choices as legitimate expressions of self-determination.

### Strengths and Limitations

This study demonstrates methodological innovation by triangulating interview data and OHC text discourse from Chinese patients with cancer, thereby enhancing the credibility and generalizability of the findings. Consistent prompting strategies and stringent data anonymization protocols minimized bias in data collection. Furthermore, the analytical approach itself represents a key strength. By using a form of triangulation—combining traditional human interpretation with 2 distinct LLMs—this study systematically mitigated the risk of singular interpretive bias. The convergence of themes across these different methods bolstered the validity of our core findings. In contrast, the unique themes identified by each approach ensured a more comprehensive and multifaceted understanding than any single method could have yielded.

Notwithstanding these strengths, several limitations should be noted. First, the interview sample was small and drawn from a single tertiary hospital in Shanghai, a major metropolitan area with advanced health care infrastructure. This sampling approach may limit the transferability of interview findings to patients in secondary hospitals, rural areas, or regions with different health care resources. To partially mitigate this limitation, we supplemented interview data with online discourse from 2 platforms that attract users from diverse geographic locations, though the online sample also skews toward users with internet access and digital literacy.

Second, the interview inclusion criteria systematically excluded patients who were more physically compromised or had lower educational attainment. This is particularly relevant given that “lack of cognition” emerged as a barrier to trial participation. Our findings regarding cognitive and informational barriers reflect the perspectives of relatively functional, educated patients; individuals with lower health literacy or more advanced disease may face even more pronounced challenges. The conclusions regarding cognitive factors should therefore be understood as applicable primarily to patients meeting our inclusion criteria, and future research should specifically target underrepresented populations.

Third, the online data were collected from 2 Chinese platforms, and the final dataset of 219 comments, while sufficient for qualitative analysis, represents a subset of the broader online discourse. The findings should be interpreted within this scope rather than as representative of all online discussions of patients with cancer.

Fourth, the cross-sectional nature of this study captures attitudes at a single point in time. Patients’ willingness to participate in clinical trials may evolve as their disease progresses, treatment options change, or new information becomes available. Longitudinal research would provide insights into how attitudes shift over the illness trajectory.

### Implications for Future Work

Future research should address 3 priorities. First, multicenter studies that include community hospitals and rural settings are needed to capture perspectives from patients with lower educational attainment and limited health care access—populations excluded by the inclusion criteria but likely facing more pronounced barriers. Second, methodological work comparing different LLM prompting strategies and examining intermodel reliability would establish best practices for AI-assisted qualitative analysis. Third, the thematic findings suggest targets for intervention development: decision aids to address patient concerns, communication strategies to correct misconceptions, and family-inclusive recruitment approaches warrant evaluation in prospective trials.

### Conclusion

This multistage qualitative study examined the attitudes of patients with cancer toward clinical trial participation in China using a hybrid analytical framework that combines investigator-led thematic analysis with LLM-assisted approaches. Seven core themes emerged as consistent factors shaping patients’ willingness to participate: treatment selection, financial burden relief, uncertainty about therapeutic efficacy, uncertainty about control groups, lack of cognition, misconceptions, and physician trust. These findings highlight the interplay of pragmatic, cognitive, and relational factors in decision-making. The convergence of these themes across all 3 analytical methods strengthens their validity. Method-specific findings, including “recognition of medical value” (investigator only), “insufficient clinical data” (DeepSeek R1 only), and “lack of information resource” (Gemini Pro 2.5 only), demonstrate the complementary value of integrating human interpretation with LLM efficiency. Practically, recruitment strategies should bridge knowledge gaps, acknowledge family involvement, and address regional disparities in trial access. The hybrid framework presented here offers a replicable model for qualitative health research seeking both analytical rigor and interpretive depth.

## Abbreviations

AI: artificial intelligence
LLM: large language model
OHC: online health community
